# Early successful weaning from continuous positive airway pressure in infants <32 weeks of gestation: predictors of success

**DOI:** 10.3389/fped.2025.1568891

**Published:** 2025-04-03

**Authors:** Lisa Marie Bünte, Christina Walden, Jennifer Schlecht, Benedikt Bubl, Mircea-Horia Popa-Todirenchi, Susanne Tippmann, Julia Winter, Eva Mildenberger, André Kidszun

**Affiliations:** ^1^ Division of Neonatology, Department of Pediatrics, Inselspital, Bern University Hospital, University of Bern, Bern, Switzerland; ^2^ Graduate School for Health Sciences, University of Bern, Bern, Switzerland; ^3^ Division of Neonatology, Center for Pediatric and Adolescent Medicine, University Medical Center of the Johannes Gutenberg-University Mainz, Mainz, Germany; ^4^ Division of Pediatric Epidemiology, Institute for Medical Biostatistics, Epidemiology and Informatics, University Medical Center of the Johannes Gutenberg-University Mainz, Mainz, Germany

**Keywords:** preterm infant, respiratory distress, weaning, continuous positive airway pressure, respiratory rate, prediction

## Abstract

**Aim:**

To identify demographic and clinical variables predictive of early successful weaning in preterm infants weaned off continuous positive airway pressure (CPAP).

**Methods:**

Single-center retrospective analysis of preterm infants < 32 0/7 weeks gestational age (GA) weaned off CPAP according to a standardized protocol April 2013–March 2017. Infants were categorized into (1) early successfully weaned (Early-W) if weaned at the first attempt and (2) delayed weaned (Delayed-W) if more than one attempt was necessary. Potential predictor variables were predefined. Association with Early-W was analyzed by multivariable logistic regression with model selection using the Akaike information criterion (AIC). Model performance was evaluated using the area under the receiver operating characteristic (ROC-AUC).

**Results:**

145 infants [79 (54.5%) were Early-W and 66 (45.5%) Delayed-W] with complete data sets were included in the analysis. A model of higher GA [odds ratio (OR) 1.66; 95% confidence interval (CI) 1.39, 2.03; *p* < 0.001], present patent ductus arteriosus (PDA) (OR 0.41, 95% CI 0.16, 1.04; *p* = 0.062) and higher median respiratory rate (RR) in the previous 12 h (OR 0.36; 95% CI 0.16, 0.74; *p* = 0.008) best predicted Early-W (ROC-AUC: 0.841).

**Conclusion:**

This study identified GA, PDA and median RR to predict early successful weaning in preterm infants weaned off CPAP. The predictive value of median RR in the 12 h prior to CPAP cessation is considered a novelty requiring further prospective investigation, with RR being a clinical parameter commonly employed in routine practice and practical for everyday application.

## 1 Introduction

Continuous positive airway pressure (CPAP) is the first-line treatment for respiratory distress syndrome (RDS) in most preterm infants (1, 2).

Initiation of CPAP in preterm infants with RDS has undergone extensive research, but the optimal way and time point of discontinuing CPAP remain a topic of debate, lacking sufficient evidence (3–8). Identifying the optimal time point for CPAP cessation in this clinical setting is crucial as both premature weaning and prolonged use of CPAP are associated with unfavorable outcomes (5). Premature cessation of CPAP therapy may lead to loss of functional residual capacity (FRC) and atelectasis, which could require subsequent respiratory support and recruitment effort (9). Repeated cycles of atelectasis and recruitment may promote atelectotrauma, potentially contributing to the development of chronic lung disease (10). At the same time, CPAP is associated with unfavorable outcomes such as pneumothorax, nasal trauma, discomfort, feeding and sleep problems (11–15). Hence, CPAP duration should be limited. To help guide clinicians facing this balancing act, there is a need to establish specific demographic and clinical variables predictive of an infant's readiness to successfully cease CPAP (5). Some variables have been previously reported in this context, most importantly gestational age (GA), but also patent ductus arteriosus (PDA) and duration of mechanical ventilation (MV) (16–18). However, the evidence is weak and sometimes contradictory, mainly comprising small observational studies with highly heterogeneous study designs. Variables predictive of an infant's readiness to cease CPAP remain a topic of controversy among clinicians in neonatology (16–22). Variables that are suitable for low-threshold use in common clinical settings appear desirable.

In this study we aimed to identify demographic and clinical variables predictive of early successful weaning in preterm infants <32 weeks GA that had been weaned off CPAP.

## 2 Methods

This was a single-center retrospective study. The study was reviewed by the local Ethics Committee of the Rhineland-Palatinate Medical Association Mainz, Germany, and a waiver of informed consent was granted [processing number 837.193.16 (10,512)]. This procedure is regulated by the state hospital law (§§ 36 and 37).

### 2.1 Participants

Infants with a GA of <32 0/7 weeks admitted to the neonatal intensive care unit (NICU) of the University Medical Center Mainz (UMC Mainz) in the 4-year period between April 2013 and March 2017 were eligible. UMC Mainz is a level III perinatal center. Infants were excluded if they: (1) did not require respiratory support, (2) did not receive CPAP after extubation, (3) received CPAP for less than six hours, (4) had a congenital malformation, or if they were: (5) out-born, (6) transferred to another unit before cessation of CPAP, (7) died before cessation of CPAP, or (8) if the medical record was not available at the time of the study. Infants were excluded if at least one criterion was met. By limiting the study population to preterm infants <32 0/7 weeks GA and applying exclusion criteria (1–4) we aimed on including preterm infants with RDS.

### 2.2 Weaning

CPAP was administered as per guideline using a starting positive end-expiratory pressure (PEEP) of 5 cm H_2_O that was escalated to 6–7 cm H_2_O if deemed necessary. PEEP reduction was commenced and conducted according to a standardized weaning protocol that had been implemented March 2013. The protocol provided for a systematic reduction of PEEP following a decision tree that was based on strict stability and failure criteria. According to the protocol, CPAP was discontinued at a PEEP of 4 cm H_2_O. For further details regarding the weaning protocol we refer to the source (23). Infants were defined as successfully weaned if they remained without positive pressure support for 72 h after cessation of CPAP therapy. The infants included in the study were categorized as (1) early successfully weaned (Early-W) and (2) delayed weaned (Delayed-W). (1) Early-W was defined as successful weaning on the first attempt of CPAP cessation. (2) Delayed-W was defined as successful weaning following two or more attempts of CPAP cessation.

### 2.3 Clinical practice for treatment of PDA

In clinical practice, hemodynamically relevant PDA was treated in mechanically ventilated preterm infants until extubation was possible. PDA was not treated in preterm infants receiving noninvasive respiratory support.

### 2.4 Demographic and clinical characteristics of the cohort

Data were collected including prenatal data, birth, infant characteristics at birth, therapeutic interventions, complications, feeding, growth and length of hospitalization.

### 2.5 Predictors of weaning success

Demographic and clinical variables potentially predictive of Early-W were pre-specified and selected based on the authors' clinical experience and literature review. Factors selected were: GA, MV duration, postmenstrual age (PMA) at CPAP cessation, weight at CPAP cessation, presence of PDA at CPAP cessation irrespective of prior PDA treatment, FiO_2_ at CPAP cessation, median respiratory rate (RR) during the 12 h prior to CPAP cessation, number of desaturations <80% during the 12 h prior to CPAP cessation.

### 2.6 Statistical analysis

The analysis sample excluded infants with missing data in any of the eight potential predictor variables. Descriptive statistics were therefore based on complete cases only. The descriptive analysis encompassed a group comparison between Early-W and Delayed-W with respect to demographic and clinical variables including the potential predictor variables. Characteristics were described by appropriate statistical parameters [e.g., absolute and relative frequencies for categorical variables, means and standard deviations (SD) for continuous variables].

To study the association between potential predictor variables and Early-W several binary logistic regression models were computed. First, eight univariable models with each potential predictor as independent variable were set up. Second, one multivariable full model with all eight potential predictors as independent variables was established. Third, to identify the best model to predict Early-W, while considering the risk for overfitting, predictor variables were selected from the full model by backward selection based on the Akaike information criterion (AIC) using the step function from the R stats package. Model results are reported as odds ratios (OR) with 95% confidence intervals (CI) and *p* values.

Performance of the full and the backwards selected model were studied by receiver operating characteristic (ROC) curves in the same sample of patients. The corresponding area under the curve (AUC) values were computed using cross-validation for more robust estimates and to further avoid overfitting. All analyses were exploratory and were carried out with R version 3.6.0.

## 3 Results

**Figure 1:**
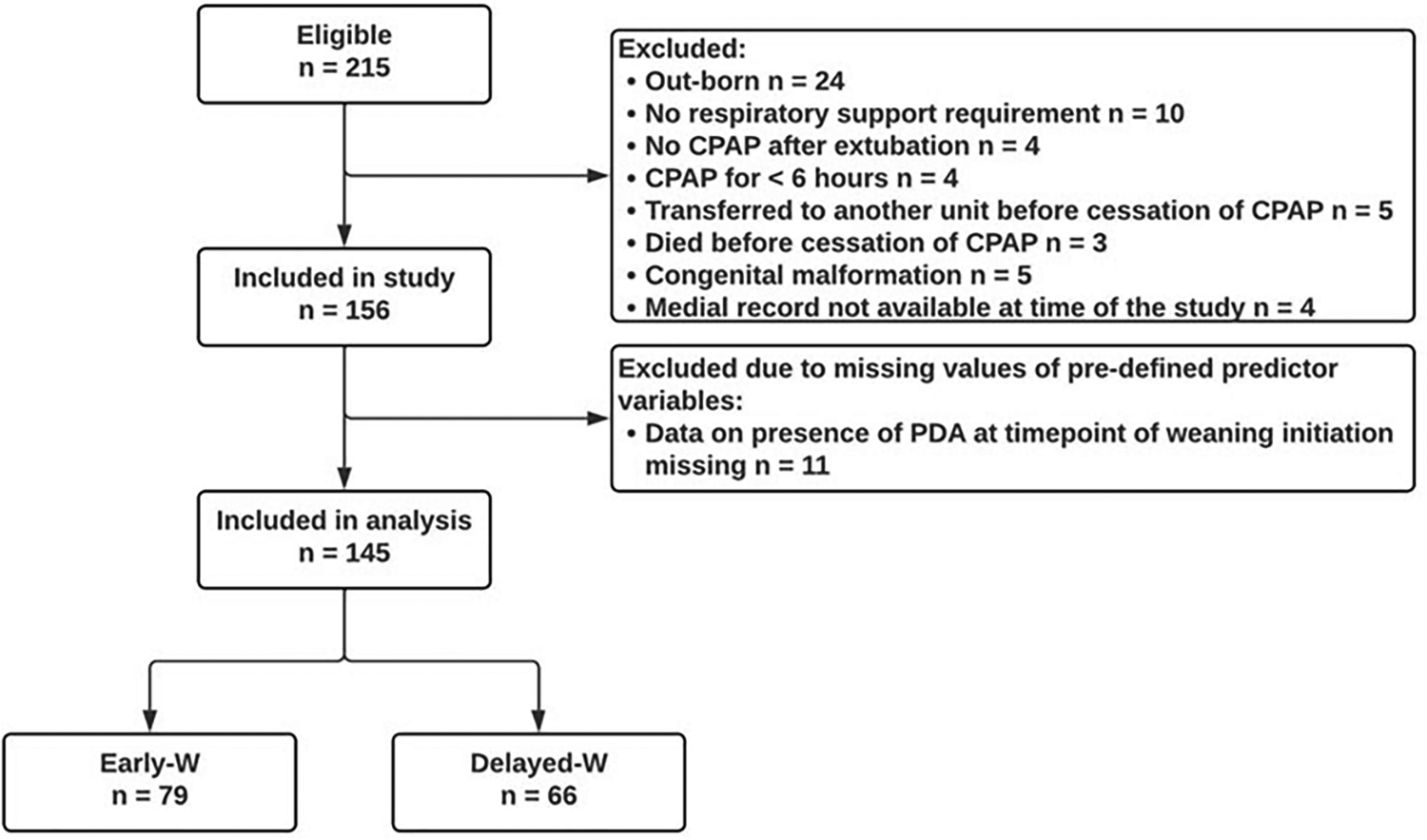
Patient flowchart for preterm infants <32 weeks GA. Abbreviations: GA, gestational age; Early-W, early successfully weaned; Delayed-W, delayed weaned; CPAP, continuous positive airway pressure; PDA, patent ductus arteriosus.

The study included 156 preterm infants. Data on the presence of a PDA at the time point of CPAP cessation was not available for 11 infants. Since the analysis sample excluded infants with missing data in any of the eight potential predictor variables, 145 infants were left for the analysis. Of these, 79 infants (54.5%) were Early-W and 66 (45.5%) were Delayed-W (see Figure 1). Early-W infants had higher GA and birth weight (BW), shorter duration of MV, received surfactant and postnatal steroids less often and had less complications related to prematurity. PMA at cessation of supplemental oxygen and CPAP were higher in Delayed-W. Table 1 summarizes the demographic and clinical characteristics of both groups. Estimates of potential predictor variables differed between groups (see Table 2).

**Table 1:** Demographical and clinical characteristics of Early-W and Delayed-W group.

Demographical and clinical characteristics	Early-W (*n* = 79)	Delayed-W (*n* = 66)	*p*-value
GA, weeks, median (IQR)	30.3 (28.8, 31.0)	26.3 (24.9, 28.1)	**<0** **.** **001** *
BW, g, mean (SD)	1,278.6 (383.2)	853.2 (302.3)	**<0** **.** **001** **
Male, %	59.5	54.5	0.666***
Singleton birth %	63.3	69.7	0.114***
Caesarean section, %	91.1	80.3	0.1***
Apgar score at 5 min, median (IQR)	8.0 (7.5, 9.0)	7.0 (7.0, 8.0)	**<0** **.** **001** *
UA pH, median (IQR)	7.3 (7.3, 7.4)	7.3 (7.3, 7.4)	0.897*
Prenatal steroids, %	98.7	97	0.591***
Postnatal steroids, %	3.8	15.2	**0** **.** **036** ***
Surfactant, %	44.3	93.9	**<0** **.** **001** ***
Caffeine citrate %	77.2	98.5	**<0** **.** **001** ***
MV duration, days, median (IQR)	0.0 (0.0, 0.7)	4.9 (1.2, 19.9)	**<0** **.** **001** *
PMA, weeks, median (IQR)			
Off FiO_2_ >0.21	31.0 (30.0, 31.6)	33.6 (31.0, 35.8)	**<0** **.** **001** *
Off CPAP	31.1 (30.3, 31.6)	32.7 (31.6, 34.0)	**<0** **.** **001** *
First oral feeding attempt	31.3 (30.3, 31.7)	30.4 (29.7, 31.7)	**<**0.161*
OFF PN	31.0 (30.1, 32.0)	28.6 (27.3, 30.3)	**<0** **.** **001** *
Discharge	38.0 (37.1, 39.4)	40.3 (38.3, 42.9)^a^	**<0** **.** **001** *
Complications, %			
Antibiotic treatment	36.7	77.3	**<0** **.** **001** ***
BPD	7.6	24.2	**0** **.** **011** ***
IVH ≥ II°	1.3	16.7	**0** **.** **002** ***
NEC, ≥5 days npo	3.8	1.5	0.626***
ROP, requiring surgery	1.3	10.6	**0** **.** **023** ***
PDA, medically treated	6.3	50.0	**0** **.** **001** ***

Note: *p*-value is written bold if significant.

Abbreviations: Early-W, early successful weaning; Delayed-W, delayed weaning; GA, gestational age; IQR, interquartile range; BW, birth weight; SD, standard deviation; UA, umbilical artery; MV, mechanical ventilation; PMA, postmenstrual age; FiO_2_, fraction of inspired oxygen; CPAP, continuous positive airway pressure; PN, parenteral nutrition; BPD, bronchopulmonary dysplasia; IVH, intraventricular hemorrhage; NEC, necrotizing enterocolitis; npo, nil per os; ROP, retinopathy of prematurity; PDA, patent ductus arteriosus.

^a^ Value missing for 1 infant.

* Wilcoxon Mann Whitney test.

** *t*-test for independent samples.

*** Chi square test, Fisher's exact test in case of <5 expected cases.

**Table 2:** Potential predictor variables in Early-W and Delayed-W group at time point of first attempt of CPAP cessation.

Potential predictor variables	Early-W (*n* = 79)	Delayed-W (*n* = 66)	*p*-value
GA, weeks, median (IQR)^a^	30.3 (28.8, 31.0)	26.3 (24.9, 28.1)	**<0** **.** **001** *
MV duration, days, median (IQR)^a^	0.0 (0.0, 0.7)	4.9 (1.2, 19.9)	**<0** **.** **001** *
PMA, weeks, median (IQR)	31.1 (30.3, 31.7)	31.3 (30.3, 32.1)	0.175*
Weight, g, median (IQR)	1,310.0 (1,155.0, 1,490.0)	1,315.0 (1,101.2, 1,522.5)	0.806*
PDA present, %	17.7	33.3	**0** **.** **048** **
FiO_2,_ median (IQR)	0.21 (0.21, 0.21)	0.21 (0.21, 0.25)	**<0** **.** **001** *
Median RR during previous 12 h, breaths/min, mean (SD)	49.4 (6.0)	54.8 (7.0)	**<0** **.** **001** ***
Number of desaturations <80% during previous 12 h, median (IQR)	1.0 (0.0, 2.5)	9.5 (2.0, 18.5)	**<0** **.** **001** *

Note: *p*-value is written bold if significant.

Abbreviations: Early-W, early successful weaning; Delayed-W, delayed weaning; CPAP, continuous positive airway pressure; GA, gestational age; IQR, interquartile range; MV, mechanical ventilation; PMA, postmenstrual age; PDA, patent ductus arteriosus; FiO_2_, fraction of inspired oxygen; RR, respiratory rate; SD, standard deviation.

^a^ Already listed in Table 1 for reference, presented here again for better clarity.

* Wilcoxon Mann–Whitney test.

** Chi square test.

*** *t*-test for independent samples.

Univariable logistic regression analysis of the eight potential predictor variables showed a significant association of six variables with Early-W. In multivariable logistic regression analysis including all eight potential predictor variables two variables remained significantly associated: GA and median RR in the 12 h prior to CPAP cessation. Backwards selection of the full model using the AIC singled out GA, PDA and median RR in the previous 12 h to be the best model to predict Early-W. (1) Each additional week of gestation increased the chance of Early-W by 66% (OR 1.66; 95% CI 1.39, 2.03; *p* < 0.001). (2) Presence of PDA at time point of CPAP cessation decreased the chance of Early-W by 59% (OR 0.41; 95% CI 0.16, 1.04; *p* 0.062). (3) Each 10 breaths/min increase of median RR over the 12 h prior to CPAP cessation decreased the chance of Early-W by 64% (OR 0.36; 95% CI 0.16, 0.74; *p* 0.008). Table 3 summarizes the logistic regression analysis for prediction of Early-W.

**Table 3:** Logistic regression analysis of potential predictor variables for Early-W, *n* = 145.

Potential predictor variables	Univariable models		Full model		Backwards selected model (Criterion: AIC)	
	OR [95% CI]	*p*-value	OR [95% CI]	*p*-value	OR [95% CI]	*p*-value
GA, per week	1.75 [1.48; 2.14]	**<0** **.** **001**	1.64 [1.19; 2.34]	**0** **.** **004**	1.66 [1.39; 2.03]	**<0** **.** **001**
MV duration, per day	0.91 [0.87; 0.95]	**<0** **.** **001**	1.03 [0.95; 1.11]	0.471	–	–
PMA, per week	0.85 [0.69; 1.04]	0.119	0.81 [0.50; 1.22]	0.340	–	–
Weight, per 100 g	1.02 [0.92; 1.12]	0.741	1.17 [0.98; 1.41]	0.081	–	–
Presence of PDA	0.43 [0.20; 0.92]	**0** **.** **032**	0.41 [0.15; 1.07]	0.071	0.41 [0.16; 1.04]	0.062
FiO_2,_ per 0.01 units	0.73 [0.59; 0.85]	**<0** **.** **001**	0.97 [0.80; 1.10]	0.659	–	–
Median RR during previous 12 h, per 10 breaths/min	0.25 [0.13; 0.46]	**<0** **.** **001**	0.36 [0.15; 0.79]	**0** **.** **014**	0.36 [0.16; 0.74]	**0** **.** **008**
Number of desaturations <80% during previous 12 h	0.88 [0.83; 0.93]	**<0** **.** **001**	0.97 [0.89; 1.04]	0.355	–	–

Note: *p*-value is written bold if significant.

Abbreviations: Early-W, early successful weaning; GA, gestational age; OR, odds ratio; CI, confidence-interval; AIC, Akaike information criterion; MV, mechanical ventilation; PMA, postmenstrual age; PDA, patent ductus arteriosus; FiO_2_, fraction of inspired oxygen; RR, respiratory rate.

**Figure 2:**
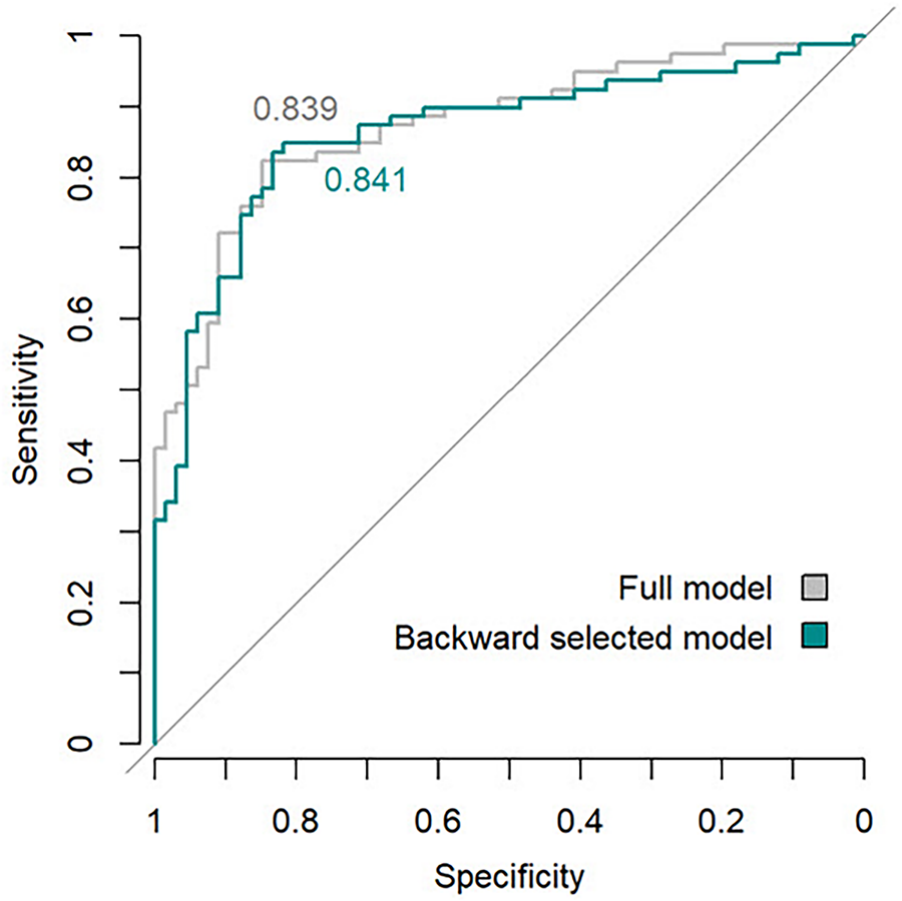
Performance of full and backwards selected model (criterion: AIC) for prediction of Early-W by means of area under ROC curves. Abbreviations: AIC, Akaike information criterion; ROC, receiver operating characteristic.

Figure 2 compares the ROC curves with respective AUC of the full model (ROC-AUC 0.839) and backwards selected model (ROC-AUC 0.841).

## 4 Discussion

We identified one demographic and two clinical variables predictive of Early-W in preterm infants with a GA <32 weeks weaned off CPAP: GA, PDA and median RR in the 12 h prior to CPAP cessation. In a prediction model containing these three variables, overall model performance in predicting Early-W was high with an AUC of 0.841. The model showed comparable performance to the full model that included all potential predictor variables.

The predictive value of median RR in the 12 h prior to CPAP cessation is considered a novelty. For every 10 breaths/min increase in the median RR over the previous 12 h, there was a 64% decreased chance of successful weaning at the first attempt (OR 0.36; 95% CI 0.16, 0.74; *p* 0.008). It is noteworthy that the predictive value of median RR in the previous 12 h remained significant in the multivariable models despite the basic group differences between Early-W and Delayed-W: Delayed-W exhibited a lower GA, lower BW, longer duration of MV, more frequent surfactant applications and postnatal steroids, and a higher incidence of complications associated with preterm birth, particularly BPD. In the regression analysis OR and *p*-value for median RR in the previous 12 h remained rather stable, indicating a strong association that persisted, even after taking the other predicting variables into account. This is particularly noteworthy with regard to the similarly strong association of GA, suggesting relatively independent effects of RR and GA. RDS in the preterm infant is caused by surfactant deficiency and lung immaturity, leading to reduced lung compliance and inability to maintain FRC (2, 24, 25). Tachypnea represents one endogenous strategy in order to maintain FRC by initiating inspiration before end of expiration (24, 25). Thus, a lower median RR in the 12 h before CPAP cessation may correspond to the ability to maintain FRC autonomously, indicating advanced maturation of the respiratory system with enhanced lung compliance. As a consequence, infants with a lower median RR would be more likely to successfully cease CPAP at the first attempt. Accordingly, one could also hypothesize a higher median RR in the 12 h before CPAP cessation to indicate insufficient respiratory support and the need for a higher PEEP than 4 cm H_2_O in those infants. Recent evidence suggests that prolonging CPAP for 2 weeks in stable preterm infants increases lung volumes at 6 months corrected age (26). However, whether increasing PEEP would be beneficial in terms of decreasing RR (i.e., improving respiratory distress) in the short term and promoting lung growth in the long term is unknown. It should be noted that RR is a clinical parameter commonly employed in routine practice, making it practical for everyday application. There seems to be disagreement on how to set the RR threshold that marks weaning failure, considering how the applied thresholds range from 60 up to 80 breaths/min (3, 4, 6, 8, 23). With regard to the estimates for median RR in Delayed-W, possibly the thresholds guiding weaning decisions should be set in the somewhat lower range.

We conclude that RR must be given greater consideration when planning adequately powered future studies that prospectively investigate the optimal time point of CPAP cessation in preterm infants <32 weeks GA. Identification of cut-off values for clinical practice must follow and may be lower than commonly expected. RR might be suitable as a steering parameter in studies that investigate the optimal PEEP-level in preterm infants <32 weeks GA receiving CPAP.

We hypothesized that preterm infants <32 weeks GA with a PDA at time point of CPAP cessation would fail weaning more often than preterm infants without a PDA (16, 18, 21). While association of PDA with Early-W was significant in the univariable model, statistical significance did not persist in the multivariable models. It seems reasonable that PDA is associated to GA and RR, the main drivers of the multivariable models, with GA and RR taking precedence over the PDA. Though, it should be noted that OR and *p*-value of the PDA only change marginally in the course of the regression analysis, indicating a trend, closely missing the threshold of significance nevertheless. It is important to note, that in the regression analysis no distinction was made between hemodynamically relevant and non-relevant PDA. However, the description of the cohort indicates 50.0% medically treated PDAs in Delayed-W vs. 6.3% in Early-W (see Table 1). Hence, one could argue that distribution of hemodynamically relevant PDA was possibly not even among infants with a PDA in both groups, though we could not test this with the available data. It is interesting that the PDA does not get rejected when selecting the best model using the AIC. Model selection using the AIC is not based on *p*-values, rather the AIC aims for a balance between best model fit and least complexity (i.e., lowest number of variables) to avoid overfitting. Considering all of the above, we conclude that, when taking GA and RR into account to predict Early-W, information on presence or absence of PDA adds to the predictive capability of the model. Further sufficiently powered studies are needed to evaluate the differential effect of PDA in the context of important demographic and clinical variables on early successful CPAP weaning in preterm infants <32 weeks GA.

The predictive value of GA has already been recognized in previous studies (16–18). Reasonably, prematurity contributes to an overall poorer health status, which particularly affects the respiratory system and leads to a higher incidence of BPD among other complications of prematurity. This results in a longer dependence on non-invasive respiratory support. Correspondingly, in our study Delayed-W had a significantly higher incidence of BPD than Early-W infants. Considering the above, individual weaning strategies tailored to GA appear beneficial. However, corresponding protocols are scarce in clinical practice (7, 27, 28).

There are limitations to our study. This was a single-center design and the sample size was relatively small. The design was retrospective, though weaning decisions during the study period were based on a standardized weaning protocol (23). The backwards selected model was trained and tested using the same patient cohort, carrying the risk of overfitting. To address this possible limitation while considering feasibility and limited sample size, AUC were calculated using cross-validation. The prevalence of Early-W was high (54.5%), therefore the calculated ORs may overestimate the relative risk. Study group distinction was based on the definition of being “successfully weaned” based on clinical stability criteria used at our study site (23). Variability of applied clinical stability criteria depending on study site is a known challenge, making our results less generalizable (3, 28). However, the observed prevalence of Early-W was comparable to similar studies (16, 17). With the inclusion of preterm infants up to a GA of <32 weeks, subgroup analysis for GA would have been desirable, yet not feasible due to limited sample size. Nevertheless, GA was included in the multivariable regression models.

## 5 Conclusion

In our study, median RR in the 12 h prior to CPAP cessation is independently predictive of Early-W. RR is a routine parameter commonly employed in clinical practice, making it practical for everyday application. RR must be given greater consideration when planning adequately powered future studies prospectively investigating the optimal time point of CPAP cessation in preterm infants. Further evidence is needed to explore the differential effect of PDA related to important demographic and clinical variables on early successful CPAP weaning in preterm infants.

## Data Availability

The raw data supporting the conclusions of this article will be made available by the authors, without undue reservation.
